# Maternal high-fat diet intensifies the metabolic response to stress in male rat offspring

**DOI:** 10.1186/s12986-017-0177-3

**Published:** 2017-02-28

**Authors:** Roxana Karbaschi, Homeira Zardooz, Fariba Khodagholi, Leila Dargahi, Mina Salimi, FatemehSadat Rashidi

**Affiliations:** 1 Neurophysiology Research Center, School of Medicine, Shahid Beheshti University of Medical Sciences, 19615-1178 Tehran, Iran; 2 Department of Physiology, School of Medicine, Shahid Beheshti University of Medical Sciences, 19615-1178 Tehran, Iran; 3 Neuroscience Research Center, Shahid Beheshti University of Medical Sciences, Tehran, Iran; 4 NeuroBiology Research Center, Shahid Beheshti University of Medical Sciences, Tehran, Iran

**Keywords:** Maternal high-fat diet, Stress, Insulin secretion, GLUT2 expression

## Abstract

**Background:**

The mother’s consumption of high-fat food can affect glucose metabolism and the hypothalamic–pituitary–adrenal axis responsiveness in the offspring and potentially affect the metabolic responses to stress as well. This study examines the effect of maternal high-fat diet on the expression of pancreatic glucose transporter 2 and the secretion of insulin in response to stress in offspring.

**Methods:**

Female rats were randomly divided into normal and high-fat diet groups and were fed in accordance with their given diets from pre-pregnancy to the end of lactation. The offspring were divided into control (NC and HFC) and stress (NS and HFS) groups based on their mothers’ diet and exposure to stress in adulthood. After the two-week stress induction period was over, an intraperitoneal glucose tolerance test (IPGTT) was performed and plasma glucose and insulin levels were assessed. The pancreas was then removed for measuring insulin secretion from the isolated islets as well as glucose transporter 2 mRNA expression and protein levels.

**Results:**

According to the results obtained, plasma corticosterone concentrations increased significantly on days 1 and 14 of the stress induction period and were lower on the last day compared to on the first day. In both the NS and HFS groups, stress reduced plasma insulin concentration in the IPGTT without changing the plasma glucose concentration, suggesting an increased insulin sensitivity in the NS and HFS groups, although more markedly in the latter. Stress reduced insulin secretion (at high glucose concentrations) and increased glucose transporter 2 mRNA and protein expression, especially in the HFS group.

**Conclusion:**

Mothers’ high-fat diet appears to intensify the stress response by changing the programming of the neuroendocrine system in the offspring.

## Background

Consuming high-fat foods is one of the environmental factors threatening the health of the embryo and the newborn. Most pregnant women tend to eat high-calorie and/or high-fat foods that affect not only their own health and pregnancy, but also supplies excessive nutrients to the embryo and may cause many diseases such as metabolic disorders in later life [1].

Many studies have shown that the mother’s consumption of high-fat food can potentially affect the neuroendocrine programming in newborns and cause glucose metabolism disorders in adulthood [2]. A chronic high-fat diet in pregnant rats increases insulin levels, impairs glucose tolerance and induces obesity in the adult offspring [1, 3]. Another study conducted on rats showed that the offspring of mothers on high-fat diets develop hyperglycemia and hyperinsulinemia and increased plasma triglyceride, body fat, liver weight, hepatic triglyceride content and vascular disorders [4–6]. Nevertheless, there are limited studies on the effect of a maternal high-fat diet on pancreatic beta-cell function. A study in mice showed that a maternal high-fat diet leads to glucose intolerance and reduces glucose-stimulated insulin secretion (GSIS) from the isolated pancreatic islets while increasing glucose transporter 2 (GLUT2) and glucokinase (GK) mRNA expressions [1]. Another study in rats showed that maternal high-fat feeding during pregnancy and/or lactation decreases plasma insulin concentrations and pancreatic GK and GLUT2 mRNA and protein expressions but increases pancreatic GLUT2 protein in the offspring at postnatal day 21, i.e. PND21 [7, 8].

Studies suggest that maternal high-fat diet acts as a psychophysical stressor that increases the plasma corticosterone concentration in the dams and changes the programming of the hypothalamic–pituitary–adrenal axis (HPA) axis in the offspring [9]. Such a diet thus appears to change the HPA response to stress in later life. Trottier et al. examined the HPA response to stress in the offspring of dams fed with high-fat foods during lactation and found that this diet reduces the adrenocorticotropic hormone (ACTH) and corticosterone response to maternal separation stress when accompanied by an exposure to ether in rats at PND11; whereas another group of rat offspring exposed to forced swim stress showed increased ACTH secretion at PND35 compared to the control group [10]. Despite the various studies showing the effect of a maternal high-fat diet (during pregnancy and lactation) on metabolic factors in the offspring (on weaning time and in adulthood) and the few conducted on the HPA response to stress in the offspring born from high-fat-fed dams, no studies have yet examined the effect of a maternal high-fat diet on offspring’s metabolic responses (especially glucose metabolism) to stress. The present study was therefore conducted to examine the effect of a maternal high-fat diet during pre-pregnancy, pregnancy and lactation on GSIS from the isolated pancreatic islets in young adult male rat offspring in response to chronic variable stress. Pancreatic GLUT2 expression and glucose tolerance were also examined.

## Methods

### Animals

The animals were kept in a temperature-controlled room (22 ± 2 °C) with a 12 h light/dark cycle (lights on at 07:00). Twenty female Wistar rats (180 ± 20 g) were randomly divided into two treatment groups and fed with either a normal diet (N) or a high-fat diet (HF) during pre-pregnancy (4 weeks), pregnancy and lactation. The normal diet (standard pellets, produced by Pars Co., an animal food manufacturer in Iran) contained 2 g% soybean oil, which provided 4.75% Kcal as fat. The high-fat diet, which was prepared by mixing 65 g% ground standard pellets with 35 g% cow butter to provide 58.2% Kcal as fat (56.12 Kcal% from cow butter and 2.08 Kcal% from soybean oil). Table 1 presents the fatty acid composition of the N and HF diets. All the rats were given food and tap water ad libitum.

**Table 1:** Fatty acid composition of the normal and HF diets

		Percent of fatty acid	
Type of fatty acid	Common name	Normal diet	HF diet
C12:0	Lauric acid	0.30	5.00
C14:0	Myristic acid	0.27	2.88
C16:0	Palmitic acid	14.4	18.50
C16:1c n-7	Palmitoleic acid	0.00	0.16
C17:0	Margaric acid	0.00	0.32
C18:0	Steric acid	3.25	3.83
C18:1c n-9	Oleic acid	32.34	32.85
C18:2c n-6	Linoleic acid	44.96	33.06
C18:3c n-6	γ-Linolenic acid	3.90	2.66
C20:0	Arachidonic acid	0.11	0.11
C20:1c n-7	Paullinic acid	0.17	0.11
C22:0	Behenic acid	0.10	0.10
C24:0	Lignoceric acid	0.10	0.07
Other	-	0.09	0.36

The female rats were mated with a control male rat. Mating was confirmed if sperm was present in the vaginal smear in the following morning. Following birth (the average number of pups per litter for the N diet-fed and HF diet-fed dams was 8.38 ± 0.86 and 9.13 ± 0.85), litter size was randomly adjusted to eight pups per litter to ensure standardized nutrition until weaning.

The number of male pups per litter in the N diet-fed and HF diet-fed dams was 3.75 ± 0.45 and 3.25 ± 0.49 and their average birth weights were 6.72 ± 0.14g and 6.34 ± 0.11g.

The postpartum death rate per litter in the maternal N diet-fed and HF diet-fed pups was 2.13 ± 0.72 and 2.25 ± 0.37.

From weaning time (PND22), the male offspring were housed separately (three per cage) and had free access to a normal diet until PND70. At PND56, the rats were divided into four groups according to the maternal diets and their adulthood exposure to stress: NC (normal diet control), NS (normal diet stress), HFC (high-fat diet control) and HFS (high-fat diet stress). Twenty four hours after the last stress session, an IPGTT was performed in fasting state (16 h) and the animals were then decapitated and their pancreas was removed for islet isolation or was frozen in a nitrogen tank and stored at −70 °C until their GLUT2 mRNA and protein levels were determined. Their abdominal fat and adrenal glands were also removed and weighed on a digital scale (AND, Japan, resolution 0.1 mg).

### Measurement of body weight and food intake

From PND56 to the end of the experiment, the body weight and food intake of the rats were measured twice a week using a digital scale (FEW, Japan, resolution 1 g). Food intake was monitored by measuring the difference between the amount of food put in the cage and the amount remaining after 24 h.

### The stress protocol

At eight weeks old, the male rat pups in the NS and HFS groups were exposed to a 14-day period of stress. The variable stress paradigm was used to prevent the rats from getting used to their induced stress (Table 2). The rats exposed to variable stressors for one hour at different times of the day (at 8–11 AM or 14–17 PM). The stressors included a Plexiglass chamber (5-cm internal diameter, 11-cm length) for restraint stress, a communication box for electric foot shock stress (1 mA, 1 Hz, 10 s every 1 min for 1 h) [11], a Plexiglass cage for overcrowding stress (8 rats/cage for 24 h).

**Table 2:** Schedule of variable stress

Days of stress	Type of stress	Time of stress
1	Electrical shocks	Morning
2	Restraint	Afternoon
3	Overcrowding	24 h
4	Restraint	Afternoon
5	Electrical shocks	Morning
6	Overcrowding	24 h
7	Electrical shocks	Morning
8	Restraint	Afternoon
9	Overcrowding	24 h
10	Restraint	Afternoon
11	Electrical shocks	Morning
12	Overcrowding	24 h
13	Restraint	Afternoon
14	Electrical shocks	Morning

### Blood sampling and corticosterone assessment

Blood samples were obtained from the young adult rats immediately after the first (day 1) and the last (day 14) exposure to stress (in a non-fasting state). Following pentobarbital (Sigma, USA) anesthesia (60 mg/kg; ip) [12] and making sure that the animals did not respond to their tail being pinched, their tails were cut and blood was collected into a microtube containing heparin (5000 IU/ml) (5 μl/ml blood) (Caspian Tamin, Iran) [13] and then centrifuged at 3000 rpm for 10 min. Their blood plasma was then separated and kept at −70 °C for measuring the corticosterone concentration.

### IPGTT

An IPGTT was performed in the animals after an overnight (16 h) of fasting. Glucose was injected intraperitoneally (2 g/kg) to them using a 20% glucose solution (Merk, Germany) and blood samples were taken after 10, 15, 30, 60, 90 and 120 min in order to measure plasma glucose and insulin concentrations [14].

### Islet isolation procedure

After performing IPGTT, the animals’ pancreas was removed for islet isolation using the collagenase technique [15]. The entrance of common bile duct to duodenum was clamped, the duct was cannulated with a polyethylene catheter (Portex Intravenous Cannula 2.5 F, 0.75 mm OD), and 10 ml of cold Hank’s buffer [containing in mM: NaCl, 137; KCl, 5.4; CaCl_2_, 1.2; MgSO_4_.7H_2_O, 0.8; Na_2_HPO_4_.2H_2_O, 0.3; KH_2_PO_4_, 0.4; NaHCO_3_, 4.2 (Merck, Germany)] in which collagenase P (0.45 mg/ml, Roche, Germany) was diluted was gently injected into the duct. The inflated pancreas was removed and cleaned from any non-pancreatic tissue. Then the pancreas was placed into a 50 ml falcon tube and incubated in a 37 °C water bath for 17 min. Digestion was terminated by adding cold Hank’s solution up to 40 ml. The tube was shaken for 1 min and the suspension was dispensed into a glass container. Cold Hank’s solution was added and the supernatant was aspirated after precipitation (a process which was repeated three times). After the last aspiration, the islets were handpicked (Blue Light stereomicroscope, USA) [16].

### Glucose-stimulated insulin secretion study

Insulin secretion was assessed at 5.6 and 16.7 mM glucose concentrations from the isolated islets of each animal. Five groups of ten islets for each glucose concentration were picked and placed in plastic cups (20 cups in total for each condition). After removing the excess Hank’s solution, 1 ml of Krebs-Ringer solution (pH 7.4) [containing in mM: NaCl, 111; KCl, 5; MgCl_2_.6H_2_O, 1; CaCl_2_.2H_2_O, 1; NaHCO_3_, 24 (Merck, Germany); Hepes, 10 (Sigma, USA) and BSA, 0.5 g/dl (Sigma, USA)] containing 5.6 or 16.7 mM glucose was added to the cups and incubated for 90 min (at the beginning, the cups were gassed with CO_2_ 5%/O_2_ 95% for 5 min) at 37 °C. The supernatant was then removed and stored at −70 °C for insulin assays [16].

### The assessment of plasma glucose, insulin and corticosterone concentrations

The plasma glucose concentration was determined using the glucose oxidase method (Pars Azmoon, Iran). A rat insulin ELISA kit (Mercodia, Sweden) and a corticosterone ELISA kit (DRG, Germany) were used to measure plasma insulin and corticosterone concentrations.

### Real-time quantitative reverse transcription PCR (qRT-PCR)

GLUT2 relative gene expression was analyzed using real-time qRT-PCR in three animals per group. Total RNA was isolated from the pancreas tissue (30 mg) using a total RNA purification mini kit (Favorgen, Taiwan). To eliminate the possibility of genomic DNA contamination, the RNA samples were treated with DNase 1, RNase-free (CinnaGen, Iran). The RNA quantity was determined at 260 nm (Nanodrop 2000 spectrometer, Thermo Scientific) and the RNA purity was confirmed by checking the absorbance ratio at 260:280 nm. First-strand complementary DNA (cDNA) was synthesized from 0.3 μg total RNA using a first strand cDNA synthesis kit (Vivantis, Malaysia) with OligodT primers.

The synthesized cDNA was analyzed by ABI 7500 Fast Real-Time PCR System using SYBR Green PCR Master Mix (Applied Biosystems, USA). Primers specific to GLUT2 (For; 5′-CACCAGCACATACGACACCAGAC3′, Rev; 5′-GGACACAGACAGAGACCAGAGCAT-3′) and β-actin as the reference gene (For; 5′- TCTATCCTGGCCTCACTGTC-3′, Rev; 5′-AACGCAGCTCAGTAACACTCC-3′) were used. Each sample was run in duplicate and no-template control was run with any of the primer pairs in order to check for primer dimers and reagent contamination.

The thermal cycle for amplification consisted of an initial denaturation at 95 °C for 10 min followed by 40 cycles of 15 s at 95 °C, 30 s at 61 °C and 30 s at 72 °C. Following amplification, a melt curve analysis was performed from 65 to 95 °C with 0.5 °C increments every 10 s. The qRT-PCR products were separated on 1.5% agarose gel in 1 × Tris-borate Ethylenediaminetetraacetic acid (EDTA) buffer and stained with ethidium bromide solution (CinnaGen, Iran) to further confirm the absence of non-specific amplification products.

The quantitative analysis was performed by measuring the average Cycle of Threshold (CT) values of duplicates and relative expression of GLUT2 gene transcripts was calculated by the ΔΔCT method and converted to relative expression ratio (2^(−ΔΔCT)) for statistical analysis.

### Western blot

The pancreatic samples were homogenized in lysis buffer and centrifuged to remove the cell debris. The supernatant was collected and used to quantify the total protein concentration using the Bradford method [17]. The proteins were loaded and electrophoresed on polyacrylamide gels containing sodium dodecyl sulfate (SDS) 12% and then transferred to a polyvinylidene fluoride membrane. The membranes were incubated overnight (at 4 °C) with the rabbit polyclonal GLUT2 antibody (Santa Cruz Biotechnology Inc.). The next day, the membranes were incubated for 90 min at room temperature with the secondary antibody horseradish peroxidase-conjugated goat anti-rabbit IgG (Santa Cruz Biotechnology Inc.) and were then visualized using ECL advance kit (Amershom Bioscience, USA). The quantification of the results was performed with a densitometry scan of the films, and data analysis was performed using Image J.

### Statistical analysis

All the data are expressed as the mean ± SEM. A repeated-measures analysis of variance (ANOVA) was performed in SPSS-19 (time was taken as a repeated factor and diet and stress as the independent factors). The two-way ANOVA (diet and stress taken as the factors) and the three-way ANOVA (diet, stress and glucose concentrations taken as the factors) were performed along with LSD post-hoc tests. P-values below 0.05 were considered to be statistically significant.

## Results

### Plasma corticosterone concentrations and adrenal glands weight

**Fig. 1:**
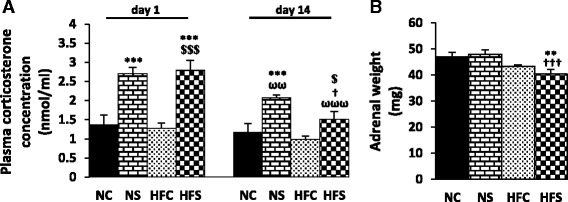
The effect of maternal high-fat diet and variable stress on plasma corticosterone concentration (**a**) and adrenal glands weight (**b**). On days 1 and 14 of the stress induction period, plasma concentrations of corticosterone were measured and the weight of the adrenal glands was determined 24 h after the last stress exposure. Each column denotes mean ± SEM (*n* = 8 per group); NC: Normal diet Control, NS: Normal diet Stress, HFC: High-fat diet Control, HFS: High-fat diet Stress; ^**^ *P* < 0.01, ^***^ *P* < 0.001 vs. NC, ^**$**^ *P* < 0.05, ^**$$$**^ *P* < 0.001 vs. HFC, ^**†**^ *P* < 0.05, ^**†††**^ *P* < 0.001 vs. NS, ^**ωω**^ *P* < 0.01, ^**ωωω**^ *P* < 0.001 vs. day 1 of the same group

Plasma corticosterone concentrations showed an increase immediately after the first and last exposure to stress in the NS (*P* < 0.001) and HFS (*P* < 0.001 and *P* < 0.05) groups compared to the control groups. A significant reduction in plasma corticosterone concentration was observed on day 14 compared to day 1 of the exposure to stress (*P* < 0.01 in the NS group, *P* < 0.001 in the HFS group) (Fig. 1a). With the exception of the HFS group, no significant changes were observed in adrenal glands weight in any of the groups. The adrenal glands weighed less in the HFS group than in the NC (*P* < 0.01) and NS (*P* < 0.001) groups (Fig. 1b).

The F values of the repeated measures ANOVA for plasma corticosterone concentration and the two-way ANOVA for adrenal glands weight are as follows:

**Table:** 

**Factors**	**Plasma corticosterone concentration**
**Time**	F(1,1) = 33.199; P = 0.000
Time * Diet	F(1,1) = 3.316; P = 0.079
Time * Stress	F(1,1) = 11.542; P = 0.002
Time * Diet * stress	F(2,1) = 1.741; P = 0.198

**Table:** 

**Factors**	**Adrenal glands weight**
**Diet**	F(3,24) = 13.454; P = 0.001
**Stress**	F(3,24) = 0.445; P = 0.510
Diet * stress	F(1,16) = 1.645; P = 0.210

### Body weight

**Fig. 2:**
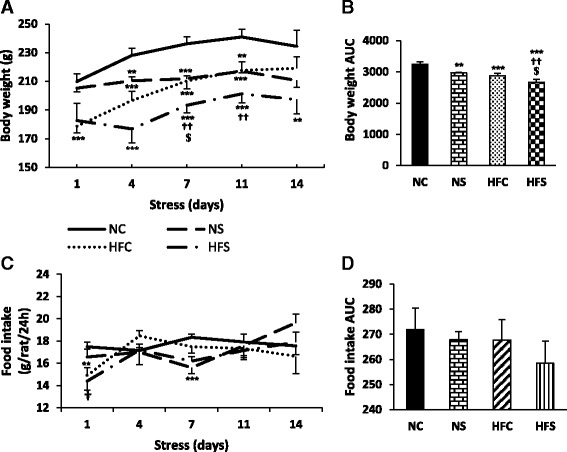
The effect of maternal high-fat diet and variable stress on body weight (**a**), body weight AUC (**b**), food intake (**c**) and food intake AUC (**d**). Body weight and food intake of the study animals were measured during the stress induction period. Each column or point denotes mean ± SEM (*n* = 8 per group); NC: Normal diet Control, NS: Normal diet Stress, HFC: High-fat diet Control, HFS: High-fat diet Stress, AUC: Area under the curve; ^**^ *P* < 0.01, ^***^ *P* < 0.001 vs. NC, ^†^ *P*<0.05, ^††^ *P*<0.01 vs. NS, ^$^ *P* < 0.05 vs. HFC

Before applying stress (on day 1), the animals weighed significantly lower in the HFC and HFS groups than in the NC and NS groups (*P* < 0.001). During the stress induction period, the offspring weighed less in the HFC group compared to the NC group on days 1, 4 and 7 (*P* < 0.001) and 11 (*P* < 0.01). The animals weighed less in the NS group compared to the NC group on days 4 (*P* < 0.01) and 7 and 11 (*P* < 0.001), but not on day 1 of the induction of stress (Fig. 2a). Nonetheless, a significant weight loss was observed in the HFS group compared to the HFC group only on day 7 of the stress induction period (*P* < 0.05). The HFS group weighed less than the NC group on days 4, 7 and 11 (*P* < 0.001) and 14 (*P* < 0.01) (Fig. 2a). The area under the curve (AUC) shows that the animals weighed less in the HFC and HFS (*P* < 0.001) and NS (*P* < 0.01) groups than in the NC group. The animals in the HFS group had a lower weight than the HFC (*P* < 0.05) and NS (*P* < 0.01) groups (Fig. 2b).

### Food intake

Before the induction of stress (on day 1), the amount of food intake was significantly lower in the HFC (*P* < 0.01) and HFS (*P* < 0.05) groups than in the NC and NS groups, while no significant differences were observed between the groups on the other days of this period (Fig. 2c). The amount of food intake was lower in the NS and HFS groups compared to the NC group (*P* < 0.001) only on day 7 (Fig. 2c). The area under the curve shows no significant differences in the amount of food intake between the groups examined (Fig. 2d).

The F values of the repeated measures ANOVA for body weight and food intake and the two-way ANOVA for their AUC are as follows:

**Table:** 

**Factors**	**Body Weight**	**Food intake**
**Time**	F(1,4) = 20.797; P = 0.000	F(1,4) = 3.601; P = 0.008
Time * Diet	F(1,4) = 6.463; P = 0.000	F(1,4) = 2.177; P = 0.076
Time * Stress	F(1,4) = 5.596; P = 0.000	F(1,4) = 4.175; P = 0.003
Time * Diet * stress	F(2,4) = 0.419; P = 0.795	F(2,4) = 0.776; P = 0.543

**Table:** 

**Factors**	**Body weight AUC**	**Food intake AUC**
**Diet**	F(3,24) = 19.604; P = 0.000	F(3,24) = 0.805; P = 0.377
**Stress**	F(3,24) = 11.038; P = 0.002	F(3,24) = 0.770; P = 0.388
Diet * stress	F(1,16) = 0.177; P = 0.677	F(1,16) = 0.129; P = 0.723

### Plasma glucose and insulin levels during the IPGTT

**Fig. 3:**
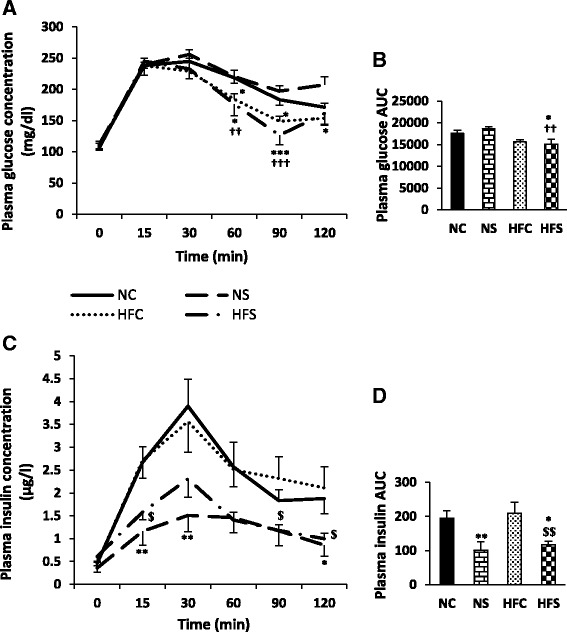
Maternal high-fat diet and variable stress effects on plasma levels of glucose (**a**) and insulin (**c**), glucose AUC (**b**) and insulin AUC (**d**) during IPGTT in young adult offspring. The glucose tolerance test was performed 24 h after the last stress exposure. Each column or point denotes mean ± SEM (*n* = 8 per group); NC: Normal diet Control, NS: Normal diet Stress, HFC: High-fat diet Control, HFS: High-fat diet Stress, AUC: Area under the curve; ^*^ *P* < 0.05, ^**^ *P* < 0.01, ^***^ *P* < 0.001 vs. NC, ^**††**^ *P* < 0.01, ^**†††**^ *P* < 0.001 vs. NS, ^**$**^ *P* < 0.05 ^**$$**^ *P* < 0.01 vs. HFC

There were no significant differences between the study groups in terms of plasma glucose concentration and insulin level at time zero (in the fasting state); however, after the intraperitoneal injection of glucose and the subsequent increase in its plasma concentrations, these values began to drop in all the groups after 30 min and approached their time zero value within 90 min (except in the NC group, which approached this point within 120 min) (Fig. 3a). The area under the curve showed that glucose concentrations were lower in both the HFC (non-significantly) and HFS groups compared to the NS (*P* < 0.01) and NC (*P* < 0.05) groups (Fig. 3b).

Plasma insulin levels increased after the increase in plasma glucose concentration during the IPGTT and reached their peak within 30 min and then gradually decreased until minute 120 (Fig. 3c). Meanwhile, plasma insulin levels were lower in the NS (*P* < 0.01 for minutes 15 and 30 and *P* < 0.05 for minute 120) and HFS (*P* < 0.05 for minutes 15, 90 and 120) groups than the NC and HFC groups. The HFS group showed no significant differences with the NC group at any time except on minute 15 (*P* < 0.05) (Fig. 3c). The area under the curve showed that stress caused a significant reduction in insulin levels in both the NS (*P* < 0.01 compared to the NC group) and HFS (*P* < 0.01 compared to the HFC and *P*<0.05 compared to the NC) groups (Fig. 3d).

The F values of the repeated measures ANOVA for plasma glucose and insulin levels and the two-way ANOVA for their AUC are as follows:

**Table:** 

**Factors**	**Plasma glucose**	**Plasma insulin**
**Time**	F(1,5) = 136.568; P = 0.000	F(1,1) = 33.436; P = 0.000
Time * Diet	F(1,5) = 6.345; P = 0.000	F(1,1) = 0.183; P = 0.969
Time * Stress	F(1,5) = 1.240; P = 0.294	F(1,1) = 5.154; P = 0.000
Time * Diet * stress	F(2,5) = 0.792; P = 0.557	F(1,1) = 1.051; P = 0.390

**Table:** 

**Factors**	**Plasma glucose AUC**	**Plasma insulin AUC**
**Diet**	F(3,24) = 13.644; P = 0.001	F(3,24) = 0.434; P = 0.516
**Stress**	F(3,24) = 0.150; P = 0.702	F(3,24) = 15.005; P = 0.001
Diet * stress	F(1,16) = 1.221; P = 0.279	F(1,16) = 0.000; P = 0.991

### Glucose-stimulated insulin secretion from the isolated pancreatic islets

**Fig. 4:**
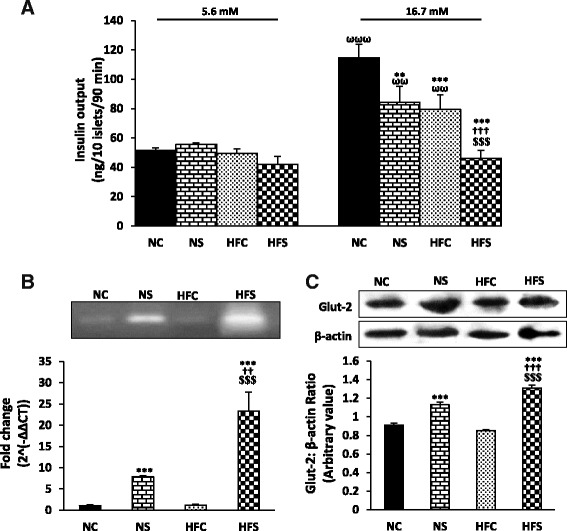
The combined effect of maternal high-fat diet and variable stress on GSIS (**a**), pancreatic GLUT2 mRNA expression (**b**) and protein amount (**c**) in the young adult offspring. Insulin secretion from the isolated islets in response to 5.6 and 16.7 mM glucose is shown (*n* = 8 per group) (**a**). Representative bands for the end product of RT-PCR are shown above the fold change (*n* = 3 per group) (**b**). Representative bands are shown above the respective densitometric values (*n* = 3 per group) (**c**). Each column denotes mean ± SEM; NC: Normal diet Control, NS: Normal diet Stress, HFC: High-fat diet Control, HFS: High-fat diet Stress, ^**^ *P* < 0.01, ^***^ *P* < 0.001 vs. NC, ^††^ *P* < 0.01, ^**†††**^ *P* < 0.001 vs. NS, ^**$$$**^ *P* < 0.001 vs. HFC, ^ωω^ *P*<0.01, ^**ωωω**^ *P* < 0.001 vs. 5.6 mM of the same group

The amount of insulin secreted in response to 16.7 mM of glucose was higher than the amount secreted in response to 5.6 mM of it in all the groups except the HFS group (*P* < 0.001 for the NC group and *P* < 0.01 for the NS and HFC groups) (Fig. 4a). There were no significant differences between any of the groups in the amount of insulin secreted from the isolated pancreatic islets in response to 5.6 mM of glucose; however, 16.7 mM of glucose in the maternal high-fat diet reduced insulin secretion by itself in the HFC group compared to the NC group (*P* < 0.001). Stress also reduced the amount of insulin secretion from the islets in both the NS and HFS groups compared to the control groups (*P* < 0.01 in the NS group, *P* < 0.001 in the HFS group). The maternal high-fat diet combined with stress in the HFS group led to a greater reduction in the amount of insulin secretion from the islets compared to the NC and NS groups (*P*<0.001) (Fig. 4a).

### Pancreatic GLUT2 mRNA expression and protein amount

In and by itself, the maternal high-fat diet did not lead to a significant change in GLUT2 mRNA expression and protein level in the HFC group compared to the NC group (Fig. 4b-c). Stress increased GLUT2 mRNA and protein expression in the NS group compared to the control group (*P* < 0.001) (Fig. 4b-c). Moreover, GLUT2 mRNA expression was significantly higher in the HFS group compared to the NC (*P* < 0.001) and NS (*P* < 0.01) groups (Fig. 4b). The amount of GLUT2 protein in the HFS group was also markedly higher compared to the NC and NS groups (*P* < 0.001) (Fig. 4c).

The F values of the three-way ANOVA for insulin output in response to different glucose concentrations and the two-way ANOVA for GLUT2 mRNA expression and protein amount are as follows:

**Table:** 

**Factors**	**Insulin output**
**Diet**	F(3,24) = 19.476; P = 0.000
**Stress**	F(3,24) = 12.412; P = 0.001
**Glucose**	F(3,24) = 42.885; P = 0.000
Diet * Stress	F(1,16) = 0.347; P = 0.558
Diet * Glucose	F(1,16) = 9.930; P = 0.003
Stress * Glucose	F(1,16) = 9.008; P = 0.004
Diet * stress * Glucose	F(2,56) = 0.077; P = 0.783

**Table:** 

**Factors**	**GLUT2 mRNA expression**	**GLUT2 protein amount**
**Diet**	F(3,9) = 35.501; P = 0.000	F(3,9) = 7.858; P = 0.023
**Stress**	F(3,9) = 122.495; P = 0.000	F(3,9) = 206.281; P = 0.000
Diet * stress	F(1,6) = 34.469; P = 0.000	F(1,6) = 24.531; P = 0.001

## Discussion

Undesirable changes in the environment in which the embryo and newborn grow and live can lead to short and long-term disorders in later stages of life. The present study was conducted to examine the effect of maternal high-fat diet as an environmental factor affecting glucose metabolism in response to stress in young adult male offspring. To eliminate the effect of the cyclic variations of female sex hormones, especially the effect of estradiol on the response to stress [18], only the male pups were studied.

Plasma corticosterone concentration was significantly higher in the stressed groups compared to in the control groups immediately after the first and last induction of stress. Just as in other studies which had used the variable stress paradigm [19, 20], the multiplicity of stressors used in this study did not lead to an adaptation in the corticosterone response to stress over time [21]. Nonetheless, plasma corticosterone concentration was lower in the HFS group compared to the NS group upon the last induction of stress. Studies have shown that, with exposure to stress, the sensitivity of the HPA axis changes in the early stages of life in the offspring. Studies have also shown that handling newborn rat offspring creates persistent changes in the sensitivity of the HPA axis to different stressors in adulthood [22, 23]. Reports suggest that a maternal high-fat diet acts like a psychophysical stressor and increases plasma corticosterone concentration in dams and changes the programming of the HPA axis in the offspring [9]. Injecting exogenous corticosterone to adrenalectomized dams has also been shown to change the functioning of the HPA axis in newborn rats [23] and intrauterine exposure to synthetic corticosterone has been shown to reduce the activity of the HPA axis in non-stimulated conditions in humans [24]. The HPA axis activity may have therefore changed in the HFS offspring in the present study due to a prenatal and postnatal exposure to a high-fat diet and a high maternal corticosterone concentration [23, 25, 26].

The adrenal glands weight showed no significant increase in the NS group and actually decreased in the HFS group. In other studies, the induction of variable stress in ten-day [27] or eight-week [28] periods increased adrenal glands weight. The disparity of results regarding this change may be due to the type of stressors used or the duration of stress induction.

Moreover, since growth factors, especially insulin-like growth factor 1 (IGF-1), have a stimulatory effect on the proliferation of different layers of the adrenal glands [29], and since a maternal high-fat diet [30] and exposure to increased glucocorticoids in prenatal and postnatal days [29, 31, 32] can reduce this factor, the lower weight of the glands in the HFS group can be due to the mutual effects of the maternal high-fat diet and corticosterone exposure on the offspring.

In line with the present findings, other studies have also shown that chronic stress in rats reduces body weight or impedes its significant increase over time compared to other rats not undergoing stress [12]. This effect is caused by alterations in the hypothalamic expression of orexigenic or anorexigenic peptides as a result of corticosterone increase due to stress exposure, which can reduce food and calorie intake [12]. Regarding the reduction in food intake in the offspring caused by maternal high-fat diet, the lower body weight in the HFS group may be indicative of the mutual effects of stress and the maternal high-fat diet.

The maternal high-fat fed offspring showed increased insulin sensitivity inapposite to the elevated insulin resistance in their mothers, as also reported in the researchers’ previous study [26]. Following stress exposure, a degree of insulin sensitivity was also observed in both stressed groups. Nevertheless, due to the faster reduction in plasma glucose concentration in the HFS group compared to the NS group, the former appears to have developed a higher insulin sensitivity. In line with the present findings, a high-fat diet rich in saturated fatty acids in rats from two weeks before pregnancy until the end of lactation increases insulin response in the adult offspring [33]. In the present study, the percentage of saturated fatty acids (30.88%) in the high-fat diet was higher than that of the normal diet (18.53%); given the importance of balanced fatty acids in diets [34] and their role in the structural and functional programming of the cells [35, 36], the increase in insulin sensitivity in the offspring may be because of the newborns’ metabolic and neuroendocrine malprogramming [9].

In line with the present findings, the use of variable stress improved glucose tolerance in adult male pre-diabetic Long-Evans rats [37]. Moreover, by administering 0.85 or 4.3 mg/day of corticosterone to chickens, Simon showed that lower corticosterone concentrations improve glucose tolerance [38]. In contrast, the induction of variable restrictive stress in rats impaired glucose tolerance [39]. In addition, most studies have shown that the constant stimulation of the HPA axis following chronic stress leads to increased blood glucocorticoid concentrations and central adiposity, insulin resistance, hyperlipidemia and hyperglycemia [40]. Based on the discussed studies, the improved glucose tolerance in the present study may be due to two factors: The type of stress induced and the decreased plasma corticosterone concentration (which was observed on the last day). Nevertheless, the mechanism by which insulin sensitivity has improved upon exposure to stress is unknown. Moreover, the better glucose tolerance in the HFS group compared to the NS group may be due to the maternal high-fat diet given during the embryonic and/or neonatal development stages [9, 35, 41] and the exposure to stress during adulthood.

In line with the present findings, insulin secretion from the isolated pancreatic islets reduced in mice with a glucose concentration of 16.7 mM that was induced by a high-fat diet during pregnancy and lactation; however, no changes occurred with a 2.8 mM glucose concentration. Moreover, GLUT2 and GK mRNA expression increased and VAMP2 (the protein responsible for insulin exocytosis) expression decreased; this reduction may be the reason for the reduction in insulin secretion in this group [1]. Another study showed that maternal high-fat diet during pregnancy and/or lactation reduced plasma insulin concentration as well as GK protein and mRNA expression in the pancreatic tissue [7].

Gremlich et al. showed that a high-fat diet reduces GLUT2 mRNA and protein expression in the pancreatic beta cells of adult rats [42], while the rats born to mothers on high-fat diets during pregnancy and lactation showed a reduced GLUT2 mRNA expression and an increased GLUT2 protein expression [7].

Given the lack of changes in GLUT2 mRNA and protein expression, the reduced secretion of insulin from the islets is perhaps caused by the disruptions in the signaling pathways involving the glucose sensors or insulin release from the beta cells. This disruption may be caused by the changes in the programming of the expression of some of the genes and proteins involved in these signaling pathways as a result of the developing embryo’s exposure to high amounts of free fatty acids in the dam’s blood in the high-fat diet groups [9, 35]. The lipotoxic and inflammatory effects of these free fatty acids in the beta cells can disrupt the cells’ normal functioning [43, 44]. Moreover, in spite of the reduced insulin secretion from the islets in the presence of 16.7 mM of glucose in the HFC group, the insulin concentration during IPGTT showed no significant differences with the control group, which may be due to the reduced insulin clearance because of the reduced expression of insulin-degrading enzyme (IDE), which is induced by the maternal high-fat diet [45] and the subsequent inflammation in the offspring.

The results of the present study showed that stress has no significant effects on the amount of insulin released from the islets in the presence of 5.6 mM glucose, which is compatible with fasting plasma insulin concentration. At 16.7 mM of glucose, stress reduced insulin secretion from the islets, which is in line with the in-vivo results obtained from the IPGTT. There are different reports on the effect of chronic stress on insulin release from the isolated pancreatic islets in the presence of basal and high glucose concentrations [16, 46–48]. The disparity of findings may be due to the differences in the type and duration of exposure to stress. Furthermore, there are limited studies on the effect of stress on GLUT2 expression in the pancreatic islets. Sadeghi et al. showed that stress in young adult rats who have also been under stress during their weaning time reduces GLUT2 levels, while being under stress only during adulthood increases the amount of this protein [46]. Another study showed that the administration of synthetic glucocorticoids such as Dexamethasone to rats for five days increases GLUT2 expression [49]. Meanwhile, the application of dexamethasone in pancreatic islet culture media was found to break down GLUT2 but have no effect on the protein’s mRNA expression [42]. The disparity of findings may be due to the differences in research methods (in vivo vs. in vitro) and the type and duration of exposure to stress or glucocorticoid.

In the presence of stress, despite the increase in GLUT2 mRNA and protein levels, insulin secretion from the islets did not change (5.6 mM glucose) or else decreased (16.7 mM glucose). Given that a maternal high-fat diet had no effects on GLUT2 expression and the reduced insulin release from the islets in response to high glucose concentrations in the HFC group, key factors other than GLUT2 appear to be involved in the production and/or secretion of insulin from the islets [50]. Nevertheless, the reduced insulin release in the presence of high concentrations of glucose may be due to the disrupted insulin secretion phases. Studies conducted on the role of stress hormones such as glucocorticoids have shown that glucocorticoids can create ER stress and thus increase oxidant agents and therefore disrupt insulin synthesis and the glucose signaling pathways [51] and thereby disrupt the GSIS with the poor folding of insulin [52]. The mutual effects of maternal high-fat diet and stress during adulthood lead to a greater reduction in insulin release from the islets compared to the separate effects of each individual factor, and the results obtained appear to be due to the combined reducing effects of each of the two factors on insulin release from the islets.

To conclude, a long-term high-fat diet in the mothers during the critical period of pre-pregnancy up to the end of lactation disrupted energy homeostasis and reduced body weight and food intake in the male offspring. This diet also increased insulin sensitivity and reduced insulin secretion from the islets independently of GLUT2 expression. Stress during adulthood caused a reduction in body weight and food intake in the offspring but increased insulin sensitivity in and by itself. Stress also reduced insulin secretion from the islets in spite of the increase in GLUT2 expression; however, these changes were more pronounced when the mother received a high-fat diet.

## Conclusion

The more pronounced changes induced by stress, i.e. reduced body weight, food intake and insulin secretion from the islets and increased insulin sensitivity and GLUT2 expression, in the offspring of high-fat fed mothers, indicate that maternal high-fat diet can intensify the response to stress in the offspring, likely by changing the neuroendocrine programming.

## Abbreviations

(q RT-PCR): Quantitative reverse transcriptase PCR chain reaction
ACTH: Adrenocorticotropic hormone
ANOVA: Analysis of variance
AUC: Area under the curve
cDNA: Complementary DNA
CT: Cycle of Threshold
EDTA: Ethylenediaminetetraacetic acid
GK: Glucokinase
GLUT2: Glucose transporter 2
GSIS: Glucose stimulated insulin secretion
HPA: Hypothalamic–pituitary–adrenal axis
IDE: Insulin-degrading enzyme
IGF-1: Insulin-like growth factor 1
IPGTT: Intraperitoneal glucose tolerance test
PND: Post natal day
SDS: Sodium dodecyl sulfate
